# Biosecurity risks to human food supply associated with plant-parasitic nematodes

**DOI:** 10.3389/fpls.2024.1404335

**Published:** 2024-04-30

**Authors:** Camelia Kantor, Jonathan D. Eisenback, Mihail Kantor

**Affiliations:** ^1^ Huck Institutes of the Life Sciences, Pennsylvania State University, State College, PA, United States; ^2^ School of Plant and Environmental Science, Virginia Tech, State College, Blacksburg, VA, United States; ^3^ Plant Pathology and Environmental Microbiology Department, Pennsylvania State University, University Park, State College, PA, United States

**Keywords:** plant-parasitic nematodes, biosecurity, human food supply, climate change, crop health, management strategies

## Abstract

Biosecurity in agriculture is essential for preventing the introduction and spread of plant-parasitic nematodes (PPNs) which threaten global food security by reducing crop yields and facilitating disease spread. These risks are exacerbated by increased global trade and climate change, which may alter PPN distribution and activity, increasing their impact on agricultural systems. Addressing these challenges is vital to maintaining the integrity of the food supply chain. This review highlights significant advancements in managing PPN-related biosecurity risks within the food supply chain, particularly considering climate change’s evolving influence. It discusses the PPN modes of transmission, factors increasing the risk of infestation, the impact of PPNs on food safety and security, and traditional and emerging approaches for detecting and managing these pests. Literature suggests that implementing advanced biosecurity measures could decrease PPN infestation rates by up to 70%, substantially reducing crop yield losses and bolstering food security. Notably, the adoption of modern detection and management techniques, (molecular diagnostics and integrated pest management) and emerging geospatial surveillance and analysis systems (spectral imaging, change-detection analysis) has shown greater effectiveness than traditional methods. These innovations offer promising avenues for enhancing crop health and securing the food supply chain against environmental shifts. The integration of these strategies is crucial, demonstrating the potential to transform biosecurity practices and sustain agricultural productivity in the face of changing climatic conditions. This analysis emphasizes the importance of adopting advanced measures to protect crop health and ensure food supply chain resilience, providing valuable insights for stakeholders across the agricultural sector.

## 1 Introduction

The term biosecurity, originally associated with national security and referred to as the defense against biological threats (e.g., biological weapons and bioterrorism) was first introduced in agriculture in the 1980s. It refers to the sum of risk management practices employed to defend against biological threats (Renault et al., 2021). The Food and Agriculture Organization (FAO) of the United Nations defines biosecurity as “a strategic and integrated concept that encompasses the policy and regulatory frameworks (including instruments and activities) that analyze and manage risk in food safety, public health, animal life and health, and plant life and health, including associated environmental risk” (FAO Biosecurity Toolkit and Food and Agriculture Organization of the United Nations, 2007). According to Renault et al. (2021), the first citation of “biosecurity” in PubMed was recorded in 1987. Since then, the term has been adopted by many organizations and included in strategic documents and policies across various economic sectors and scales (Renault et al., 2021; Huber et al., 2022).

The definition of biosecurity varies among organizations and countries. It has been recognized lately as a major concern at national and international levels (Inglesby and Henderson, 2012). Biosecurity evolved with another term, biosafety, where biosafety means protection of public health and the environment against accidental exposure to biological agents, while biosecurity involves prevention of human misuse and release of pathogens, toxins, and other biological materials into the environment that would ultimately end up harming humans (Belgian Biosafety Server). Generally, biosafety and biosecurity are complementary. In Europe, they are addressed separately within legislation, procedures, or other measures related to human, animal, and plant pathogens (Bielecka and Mohammadi, 2014).

The United States Department of Agriculture (USDA) defines biosecurity as “…a series of management practices designed to prevent the introduction, delivery, and spread of disease pathogens that can harm or adversely affect livestock, crops, environments, and people.” New Zealand introduced economic, cultural, and social values as part of biosecurity (Ministry for Primary Industries, Biosecurity (2025)). Promoting a biodiversity culture may be as important as conducting preventive actions, in the long run. In this paper, biosecurity refers to the measures taken, including cultural changes, to prevent, control, and manage the introduction and spread of plant-parasitic nematodes (PPNs) in the food supply chain, agronomic crops in particular.

Plants provide us with two of the most important components that we need to sustain life: food and oxygen. According to the FAO, (2019) report, plants are responsible for providing us with 98% of the oxygen we breathe and 80% of the food we consume. Approximately 40% of food crops are lost to agricultural pests, including PPNs (Palomares-Rius et al., 2021).

Nematodes are a large group of microscopic roundworms that are found in soil, water, and plant tissues, occupying almost every habitat on earth (Cobb, 1914). They are among the most important groups of organisms inhabiting the soil around plant roots, often playing a crucial role in their growth and productivity (Thorne, 1962). Nematodes have an amazing ability to adapt to a wide variety of environments with an evolutionary advantage for species’ survival and development (Bernard et al., 2017). PPNs are economically important agricultural pests that cause an estimated annual loss of USD 173 billion, globally (Gamalero and Glick, 2020; Kantor et al., 2022), a costly burden on crop production. In the context of climate change and the removal of some nematicides because of their negative environmental impacts, it is expected that yield losses caused by PPNs will increase significantly (Palomares-Rius et al., 2021). For example, some PPNs, such as the golden potato cyst nematode *Globodera rostochiensis* (Wollenweber, 1923; Behrens, 1975), caused 100% loss in potato fields. In one field in New York, where this pest was first reported in the U.S., losses caused by it were as high as 70% of the total production (Brodie and Mai, 1989; Kantor et al., 2022). Unfortunately, in many countries, the full extent of nematode damage is unknown since many growers are unaware of the presence of PPNs (Jones et al., 2013). They also lack the expertise in nematology and/or the Cooperative Extension System (CES) services available in countries like the U.S. where land-grant universities, indirectly through the USDA, provide agricultural informal education and learning activities. To limit the damage that PPNs cause to plants, it is crucial to identify them early and accurately and to understand their basic biology and life cycle (Decraemer and Hunt, 2006).

Some PPNs are capable of vectoring plant viruses by feeding on plant roots (Abd-Elgawad and Askary, 2015) and causing diseases that result in significant economic losses for growers. Disease outbreaks in the field caused by virus-transmitting nematodes fall into two groups:1) nepoviruses (transmitted by *Xiphinema*, *Longidorus*, and *Paralongidorus* species) and 2) tobraviruses transmitted by *Trichodorus* and *Paratrichodorus* species (Taylor and Robertson, 1975; McElroy et al., 1977).

Nematodes feed mainly on plant roots causing damage that impairs their ability to absorb water and nutrients from the soil (Bernard et al., 2017). As a result, nematode infestations can cause reduced crop yields, poor quality, and in some cases, complete crop failure. Yield losses caused by PPNs vary based on nematode species, population densities, host resistance, soil structure, and environmental stresses.

PPNs have been understudied as biosecurity threats until recently (Abd-Elgawad, 2014; Singh et al., 2014; Askary and Martinelli, 2015). While many pests and diseases that affect crops have been the subject of intensive research, PPNs have not received the same level of attention. This is partly because PPNs are difficult to study and monitor, given that they are microscopic, they usually reside in the soil and are reductive rather than destructive. A few PPNs attack the aerial parts of plants which can be quite damaging when the environmental conditions, especially humidity, can facilitate their movement (Decraemer and Hunt, 2006). PPNs are often overlooked because the symptoms they cause resemble other abiotic stresses, such as drought and nutrient deficiencies (Hassan et al., 2013). Additionally, given a critical worldwide under-representation of nematology knowledge (Coyne et al., 2018), many PPN species have been identified only recently, highlighting the need for increased nematology expertise, research support, and surveillance efforts to better understand the risks that they pose to crops and the food supply (Ali and Askary, 2001). Some parts of the world lack the resources and expertise to identify and fight against PPNs and thus, they are unable to respond to these serious threats to food security. For example, in many African countries, root-knot nematodes cause between 30-100% of crop losses (Murungi et al., 2014).

The food supply chain is complex, and the movement of PPNs from one location to another can take place during different stages, including transportation, storage, processing, and packaging. Furthermore, global trade has facilitated the movement of crops and food products across borders, which increases the risk of introducing nematode species to new environments. Research (Hassan et al., 2013; Singh et al., 2015; Bernard et al., 2017; Coyne et al., 2018) has shed some light on the extent of PPN infestations and their impact on crop yields and food security, emphasizing the need for increased biosecurity measures to help prevent the introduction and slow the spread of these pests. Such measures include quarantine, inspection, monitoring, and regulatory frameworks that limit the movement of plant materials across borders.

This article aims to provide an overview of the biosecurity risks associated with PPNs in the food supply chain. Specifically, it will discuss the modes of transmission of nematodes in the food supply; the factors that increase the risk of nematode infestation in crops; the consequences of nematode infestation on food safety and security; and the approaches for detecting, managing, and preventing the spread of nematodes. By providing a comprehensive understanding of the challenges posed by PPNs in the food supply chain, this article seeks to contribute to the development of effective measures to ensure food safety and security.

## 2 Overview of plant-parasitic nematodes

### 2.1 Introduction to PPNs

PPNs are microscopic non-segmented round worms that inhabit the soil and feed on plant roots causing significant crop damage worldwide. Their global distribution indicates that nematodes have been an important factor in the economy since ancient civilizations and probably an important factor in the famines that decimated certain nations or forced mass migrations of people (Thorne, 1961). Historically, PPNs have had a significant, yet often unappreciated impact on the early agriculture of the United States. These worms were partially responsible for the rapid deterioration of soils in the colonies along the Atlantic coast, often called “worn out” or “tired soil” (Thorne, 1961). PPNs can be classified based on their feeding behavior as migratory ectoparasites and endoparasites, migratory and sedentary endoparasites, and migratory and sedentary semi-endoparasites. Sedentary nematodes stay in one place, whereas migratory nematodes move to different places in the soil or their host plants. Semi-endoparasites are mobile during one part of their life cycle (juveniles and immature females) and become immobile in a fixed feeding site when they become adults (Bridge and Starr, 2007). There is also a category of PPNs that are facultative plant feeders (Aphelenchids and Tylenchids). They can feed on fungal hyphae in the soil, as well as on plant tissues (Eisenback, 2022). During their evolutionary history, PPNs developed several adaptations such as a stylet and the specialization of the esophageal gland cells (Eisenback, 2022) that allowed them to parasitize plants. Nematodes feeding on plants cause different symptoms that can be confused with nutrient deficiencies, making it difficult to diagnose because nematodes injure the roots. This interferes with the uptake of nutrients, sometimes causing very specific symptoms (e.g.dark-green interveinal greening of beech leaves [*Fagus grandifolia* Ehrh.] or chlorosis of peanut [*Arachis hypogaea* L.] leaves that can be associated with a particular nematode species (Carta et al., 2020; Eisenback, 2022). As a result, PPNs’ impact on food supply, especially on the major crops used in our diet, can be significant. Just as an example, it is estimated that nematodes cost rice (*Oryza sativa* L.) production approximately 35 billion dollars per year, 21 billion dollars in maize (*Zea mays* L.), and six billion dollars in potatoes (*Solanum tuberosum* L.), and wheat (*Triticum aestivum* L.) (Eisenback, 2022).

### 2.2 Most harmful nematode groups to plants and food crops

Many PPNs can have significant economic impacts on agriculture and horticulture. A list of the most important ones follows:

#### 2.2.1 Root-knot nematodes

*Meloidogyne* species in the genus first proposed by Göldi, in 1892 when he described *Meloidogyne exigua* Göldi, 1892 from coffee (*Coffea arabica* L.) roots in Rio de Janeiro, Brazil (Göldi, 1892; Hunt and Handoo, 2009). With a huge host range, RKNs are one of the most economically important PPNs in the world, causing significant damage to plants growing in fields, gardens, orchards, greenhouses, and as ornamental plants leading to reduced yields and quality. Thorne (1961) mentioned that RKN infestations of nursery stock and seedling plants account for more losses in this industry than all other diseases combined. They become infective during the second juvenile stage (J2), migrating and entering the root, usually near the apical meristem of host plants (Eisenback and Triantaphyllou, 1991). After they enter the roots, they move through the endoderm, establish a permanent feeding site, and form giant cells within the vascular cylinder that act as a metabolic sink producing large amounts of proteins and other molecules that benefit the juvenile rather than the plant (Eisenback and Triantaphyllou, 1991). The J2 becomes swollen and rapidly molts twice to form the non-feeding third and fourth-stage juveniles. These molt again where the adult female continues to feed, while the adult male does not feed, but instead leaves the root to find a mate. The female starts producing 500-1200 or more eggs that are deposited into a single gelatinous egg mass that protects the eggs from dehydration, parasites, and predators (Eisenback and Triantaphyllou, 1991).

The duration of the RKNs life cycle is heavily impacted by temperature. When it comes to *M. incognita* (Kofoid and White, 1912) Chitwood, 1949 affecting tomatoes (*Solanum lycopersicon* L.) at around 29°C, the adult females form within 13-15 days after the infective juvenile penetrates the roots. and begins laying eggs approximately 4-6 days later (Triantaphyllou and Hirschmann, 1960). Egg-producing females may live for 2 to 3 months, whereas the lifespan of non-feeding males is considerably shorter. RKNs under favorable conditions complete their life cycle within 4-5 weeks with some species doing so faster, in as little as 3 weeks (Bridge and Starr, 2007).

#### 2.2.2 Cyst nematodes (*Heterodera* spp. and *Globodera* spp.)

Cyst nematodes, encompassing species from the genera *Heterodera* and *Globodera*, represent a critical challenge to global agriculture due to their detrimental effects on major crops. Within the genus *Heterodera*, the species *H. glycines* Ichinoe, 1952, commonly known as the soybean cyst nematode, is a significant threat to soybean (*Glycines max* (L.) Merr. cultivation, a crop of paramount importance around the globe. This nematode species is notorious for its capacity to inflict considerable yield reductions, making its management a complex and pressing issue for farmers and agricultural professionals.

Potatoes serve as a staple food source and a key agricultural product in numerous countries underscoring the importance of addressing the challenges posed by these nematodes. The genus *Globodera* includes the potato cyst nematodes, specifically *Globodera pallida* (Stone, 1973) Behrens, 1975 and *Globodera rostochiensis* (Wollenweber, 1923) Beherns, 1975, which are recognized as major pests in potato. Similar to their counterparts in the genus *Heterodera*, potato cyst nematodes can lead to substantial yield losses, complicating their control and posing a significant threat to food security and agricultural productivity.

Species of both *Heterodera* and *Globodera* demonstrate a remarkable ability to affect the health and yield of their respective host crops, making them a focal point of research and management strategies in the quest to safeguard crop production from their damaging impacts. The difficulty in managing these pests stems from their persistent life stages and the limited effectiveness of resistant cultivars and other available control methods, highlighting the need for continued research and the development of innovative management strategies to combat these pervasive agricultural pests.

#### 2.2.3 Lesion nematode (*Pratylenchus* spp.)

Are major pests for many crops, the third most important group in terms of economic impact after RKNs and cysts (Castillo and Vovlas, 2007). They are major pests of many plants including ornamental plants, tree crops, horticultural plants, vegetables, as well as row crops (Figueiredo et al., 2022). Some species of the genus such as *Pratylenchus penetrans* (Cobb, 1917) Filipjev and Stekhoven, 1941 have a wide host range comprising over 400 plant species (Figueiredo et al., 2022). The geographic distribution of the lesion nematode is global, being reported from every continent except Antarctica. These nematodes are migratory endoparasites that cause a lot of destruction to the host root system, including brown to black lesions, cracking, internal rotting (for tubers), and weakening of the host which leads to secondary infections caused by other fungal (*Verticilium* wilt or *Fusarium oxysporum* Schltdl.) (Castillo and Vovlas, 2007) and bacterial pathogens (Sitaramaiah and Pathak, 1993). Like other PPNs, the symptoms caused by the lesion nematodes are very similar to symptoms caused by nutrient deficiencies or drought due to the disruption that these nematodes cause to normal root development and function. Root lesion nematode feeding on seedlings could lead to a poorly developed foliage and root system, which ultimately influences the plant density at harvest (Castillo and Vovlas, 2007). Many root lesion species are adapted to endure abiotic stress and some species are capable of cryptobiosis. Being able to undergo anhydrobiosis is one of the main reasons these nematodes are so difficult to eradicate (Castillo and Vovlas, 2007).

#### 2.2.4 Reniform nematodes (*Rotylenchulus reniformis* Linford and Oliveira, 1940)

These nematodes are sedentary semi-endoparasites and represent a major pest of cotton (*Gossypium hirsutum* L.), soybean, tomato, sweet potato (*Ipomoea batatas* (L.) Lam.), pineapple (*Ananas cosmosus* (L.) Merr.), papaya (*Carica papaya* L.), and numerous vegetables. They have a host range of hundreds of plant species and can persist for several months without any rain, undergoing anhydrobiosis. They cause significant yield losses to the host plants and are difficult to manage. In contrast to the RKN, the J2 goes through several molts over 7-10 days to become immature males and females (the infective stage) in the soil without feeding (Bridge and Starr, 2007). Like RKN, once the immature female starts feeding, they induce the formation of syncytia, which are functionally very similar to the giant cells induced by RKN. Males of reniform nematodes are also vermiform and develop without feeding. After fertilization, females deposit their eggs into a gelatinous matrix that surrounds the female’s body, with one egg mass containing between 50-100 eggs (Bridge and Starr, 2007).

#### 2.2.5 Citrus nematodes (*Tylenchulus semipenetrans* Cobb, 1913)

These nematodes are major pests of citrus trees, an important crop in many parts of the world. They are also major pathogens of grape (*Vitis vinifera* L.), olive (*Olea europaea* L.), loquat (*Eriobotrya japonica* [Thunb.] Lindl.), and persimmon (*Diospyros* spp.) species and cultivars (Ibrahim et al., 2022). They can cause stunted growth, and reduced yield, and can make the trees more susceptible to other diseases. The J2 of the reniform nematode enters the root and develops into mature females which will have the neck of the nematode deeply inserted in the root and establish several nurse cells in the pericycle around the head similar to the reniform nematodes (Bridge and Starr, 2007).

#### 2.2.6 Virus-transmitting nematodes (*Xiphinema* spp., *Longidorus* spp., *Paralongidorus* spp, *Trichodorus* spp., and *Paratrichodorus* spp.)

These nematodes are very important economically because they can transmit about fifteen different kinds of plant viruses from two genera of viruses, the *Nepovirus* and *Tobravirus*. It was not until 1958 when Hewitt et al. demonstrated that *Xiphinema index* could transmit the *Grapevine fanleaf virus* (GFLV) (Hewitt et al., 1958). GFLV is the most severe grapevine virus disease worldwide and can reduce grape yields by up to 80%. *Xiphinema index* is the major vector for transmitting this virus (Villate et al., 2008; Goraya et al., 2023). The global grape industry experiences significant economic losses due to this viral infection (Bernard et al., 2017). Since this discovery, the nematode group has garnered a lot of interest from a diverse array of researchers, catalyzing rapid advancements in a relatively short period. PPNs belonging to the Dorylaimida (*Longidorus* spp., *Paralongidorus* spp., *Xiphinema* spp.) can transmit *Nepovirus* while the nematodes belonging to the Trichodoridae (*Trichodorus* spp. and *Paratrichodorus* spp.) transmit tobraviruses (Hooper, 1974). Dagger nematodes are ranked eighth among PPNs in terms of causing significant damage to crops grown around the world (Jones et al., 2013; Goraya et al., 2023). Prunus stem pitting (PSP) is a lethal disease caused by the *Tomato ring spot virus* (ToRSV) to which all peach varieties and stone fruits are susceptible (Halbrendt, 2021). The main vector for its transmission is the dagger nematode. Because orchards and vineyards are long-term investments and expensive to establish, it is critical to test for the presence of dagger nematodes and ToRSV (Halbrendt, 2021). Broadleaf weeds serve as reservoirs of ToRSV and their control is essential to reducing the occurrence of this virus in the vineyard. Other crops are also vulnerable to dagger nematodes. For example, tomato yields can decrease by 50% in the presence of ToRSV (Taylor and Brown, 1997; Goraya et al., 2023).

## 3 Biosecurity risks of plant-parasitic nematodes in the food supply chain

PPNs can be a significant threat to plant health and can cause significant economic losses in agricultural and horticultural crops. When nematodes infect crops, they cause reduced yields, poor-quality produce, and, in severe cases, crop failure. As a result, biosecurity measures are essential to prevent the introduction and spread of these nematodes. Biosecurity measures can include practices such as quarantine of imported plant materials, inspection, and certification of plant materials for sale and distribution, and use of certified clean planting material. Additionally, practices such as crop rotation, soil management, and sanitation can help reduce nematode populations and prevent the spread of infestations. Nematode infestations can also affect food safety by increasing the risk of microbial contamination and reducing the effectiveness of pest management practices. In agricultural settings, integrated pest management (IPM) strategies can be employed to reduce reliance on chemical treatments and minimize the impact of nematodes on crop yields. This can include using crop varieties resistant to nematodes, using biological control agents such as nematophagous fungi and pathogenic bacteria, and employing cultural practices such as planting cover crops. It is important for growers and producers to be aware of the risks associated with PPNs and to take proactive steps to minimize their impact. This can include frequently monitoring crops for signs of nematode infestations, working with local extension offices and pest control experts, if available in the country, and staying up to date on best practices for biosecurity and pest management.

Recent outbreaks of nematodes have occurred when a pathogenic nematode was accidentally released into a new habitat or host that has not been exposed to the pathogen, making them extraordinarily vulnerable to attack. For example, *Litylenchus crenatae* Kanzaki et al., 2019 causes only minor symptoms on its host, the Japanese beech tree (*Fagus crenata* Blume) (Kanzaki et al., 2019) in Japan, but kills beech trees (*Fagus grandifolia* Ehrh.) in North America (Vieira et al., 2023). Whereas the pinewood nematode, *Bursaphelenchus xylophilus* (Steiner and Buhrer, 1934) Nickle, 1981 kills susceptible pine tree species (*Pinus densiflora* Siebold and Zucc., and *P. thunbergii* Parl.), in Japan, but peacefully coexists with the pine tree species (*Pinus banksiana* Lamb. and *P. taeda* L.) in North America.

### 3.1 Modes of transmission of nematodes in the food supply chain

**Figure 1:**
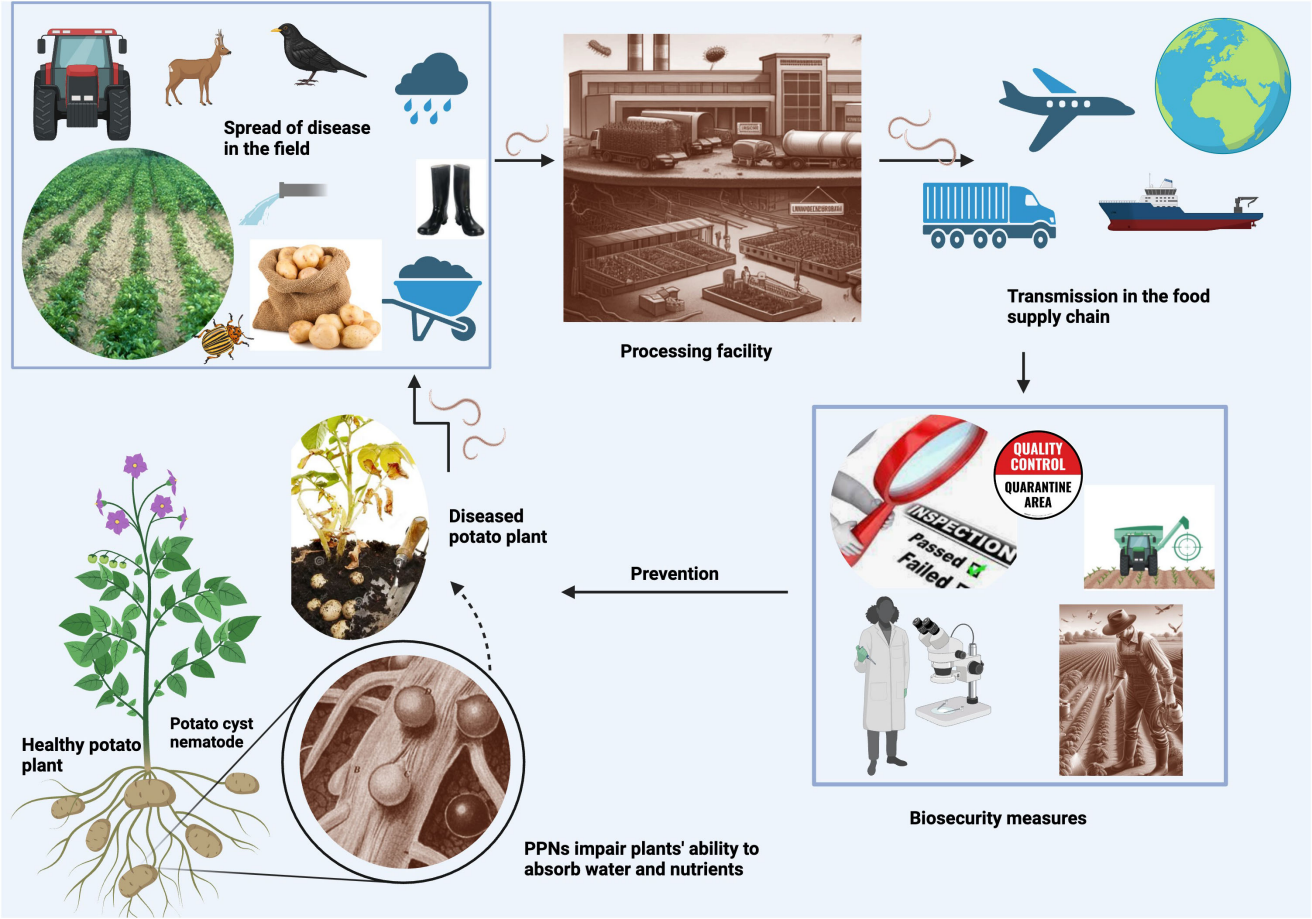
PPN transmission pathways Created with BioRender.com. This figure depicts how PPNs endanger food security, showing their infection process in plants, spread mechanisms in fields, movement through the food supply chain, and necessary biosecurity measures. It contrasts healthy and infected plants to illustrate the detrimental effects of PPNs on crop health and productivity.

PPNs can be transmitted through various pathways (**Figure 1**) in the food supply chain. Is important to understand these modes of transmission to prevent their spread. One of the most common modes of transmission is through contaminated soil. While some PPNs are migratory, moving from soil to plant tissues, most economically important PPNs are sedentary, endoparasitic or ectoparasitic, feeding on the host plant tissues (Pulavarty et al., 2021). By living inside plant tissue and limiting their movement in the soil, nematodes evade predation from bacteria, fungi, and other nematodes. PPNs do not typically move more than a meter during their lifetime unless part of contaminated materials such as soil, organic fertilizers, machinery, planting material, wind, animals, such as birds and mammals, and water movement during floods (Lambert and Bekal, 2002; Abd-Elgawad and Askary, 2015; Hartman et al., 2015; El-Saadony et al., 2021). Thus, long-distance transmission happens because of environmental factors or due to poor pest management practices which could be avoided in most cases. The best place to look for an invasive species is near the entrance point of the field.

Transmission happens when soil adheres to the surface of fresh produce, such as root vegetables, that are harvested from contaminated fields. Nematode-infested soil can also be carried by farm equipment, or muddy shoes, and transported to other fields, spreading nematodes to previously uninfected areas. For example, several species of RKNs were likely brought to North America in potted plants that were brought over from Europe by our ancestors (Neal, 1889). Nematodes can also survive in soil that adheres to packaging material, such as burlap seed bags and crates or containers used to transport produce. U.S. founding fathers, including Benjamin Franklin and Thomas Jefferson with the aid of missionaries, travelers, and state department officials brought numerous seeds and plants from many exotic locations (Shurtleff and Aoyagi, 2004). Certainly, these seeds and plants contained many unsuspecting hitchhikers.

Thus, contaminated packaging material can also spread PPNs through the soil. If it is not properly cleaned, nematodes can be spread to new areas. PPN soil infection can also lead to transmission of plant-infecting viruses as well as secondary infections with fungal or bacterial pathogens (Tileubayeva et al., 2021).

Water is an underestimated mode of transmission for PPNs, especially over long periods. While PPNs are usually terrestrial, they do live in the thin film of water surrounding soil particles. With few exceptions, they cannot infect plants directly in the water but use stored lipid reserves that allow them to survive for long periods (weeks) while maintaining their ability to infect plants (Hugo and Malan, 2010). Nematodes can be transmitted through contaminated irrigation water, water used for washing produce, rivers (especially those used for irrigation), and runoff water from infected agricultural fields (Smith and Van Mieghem, 1983). This transmission can be further exacerbated during floods. Godfrey (1923), and later Faulkner and Bolander (1966, 1970) were the first ones to draw attention to the possibility that irrigation water may be responsible for PPNs’ dispersion. Rivers, irrigation canals, runoff water, ponds, lakes, and dams seem to be the areas most prone to PPN transmission. But PPNs have also been found in less expected places such as municipal water, collected rainwater, and drainage water in soilless cultures with hydroponic-like systems (Hugo and Malan, 2010).

Plant material can also carry nematodes through soil, water, and air, and spread them to other plants. PPNs can be transmitted through infected plant material, such as seeds, seedlings, bulbs, or cuttings. This can happen when infected plant material is used to grow new crops, or when it is transported from one location to another. When quarantine officials are not careful and/or strict measures and transportation procedures are not in place, the movement of PPNs can cross country borders. Perhaps the most famous incident of imported contaminated plants is the cherry tree (*Prunus incisa* Thunb.) fiasco of the 1912 Japanese gift of friendship to the People of the United States from the People of Japan. Unfortunately, the first shipment of two thousand trees had to be destroyed because they were contaminated with RKNs and other disease agents. This prompted the formation of APHIS, the Animal and Plant Health Inspection Service which has, no doubt, prevented the introduction of many plant and animal diseases. Unfortunately, we only hear about their failures.

Humans are not the only ones to be blamed for the spread of PPNs; migrating birds can carry them along their flying paths or they can be spread by blowing winds or plant debris, especially if dry during one of their environmentally resistant stages (Lambert and Bekal, 2002).

Insects can act as passive carriers of PPNs and transmit them to new host plants (Gupta and Borges, 2021). This is because any organism feeding on infected plants and traveling between plants can potentially serve as a virus transport device. Many plant pathogens are transmitted by hemipteran insects in a circulative manner, whereby the pathogen is ingested and moves throughout the body of the insect before transmission to a new host plant (Heck, 2018). Aphids and leafhoppers, for instance, are common vectors of plant viruses and can also transmit PPNs to plants while feeding. These insects can carry nematodes from an infected plant to a new plant, thereby spreading the nematodes to previously uninfected areas.

Finally, animals can also spread PPNs. Some nematode species can infect animals, such as rodents, and can be transmitted through their feces. This can contaminate soil, water, and other areas where animals have been. PPNs can be carried by pigs (*Sus domesticus* Erxleben, 1777), cows (*Bos taurus* Linnaeus, 1758), horses (*Equus ferus caballus* Linnaeus, 1758), and birds, including domestic and migratory species.

In summary, any process that moves soil or plant tissue can disperse PPNs. It is important to understand these modes of transmission as well as factors that increase the risk of PPN infestation in crops to prevent their spread and to ensure food safety and quality within the food supply chain.

### 3.2 Factors that increase/decrease the risk of nematode infestation in crops

#### 3.2.1 Crop health

The term “crop health” is often broadly interpreted to encompass various detrimental influences on plants, whether they are biological, chemical, or physical, which can impact the physiological well-being and yield of crops. Numerous interpretations of this term have been suggested, as discussed by Döring et al. (2012). Maintaining the well-being of plants is a crucial aspect of enduring agricultural practices (Savary, 2014).

PPNs secrete effector molecules into the cells of plant roots via their stylets, thus altering root cell functions. To counter this, plants trigger their defense mechanisms against nematode infestation, detecting the intrusion through a variety of distinct and synergistic systems (Khan & Khan, 2021). Therefore, the health of a crop is a very important factor in mitigating the potential damage caused by PPNs. The resilience of crops to PPNs, such as RKNs, is significantly influenced by the health of the plant, which in turn is determined by a variety of factors. Vigorously growing crops with adequate nutrients, water, and sunlight possess robust defense mechanisms, making them more capable of withstanding PPN attacks compared to plants under stress from nutrient deficiencies, drought, or other adverse conditions. Soil quality, including its physical properties like temperature, texture, structure, and moisture, as well as chemical properties like pH and mineral composition, plays a crucial role in plant health. Plants grown in nutrient-rich soils with suitable pH and organic matter content are less prone to nematode infestations (Palomares-Rius et al., 2015), impacting both the behavior and development of RKNs (Castillo et al., 2006; Landa et al., 2014; Habteweld et al., 2024) and highlighting the interconnectedness of soil abiotic factors, plant health, and nematode behavior and development. Likewise, soil microbes can either aid the plant in its defense against PPNs, or may make the plant more susceptible (Liu et al., 2023; Zhou et al., 2024).

The approaches to managing plant health vary widely, ranging from subtle and generally broad-based strategies to more obvious and typically specialized methods extensively described in the literature (Savary, 2014).

#### 3.2.2 Climate change

Climate change can have significant impacts on the distribution and spread of PPNs, which can in turn affect food security and safety. With global warming, the geographical distribution range of PPNs could widen, spreading nematode problems to previously unaffected regions (Chakraborty et al., 2000) depending on the species involved. Climate change can lead to uneven effects, with some areas benefiting through reduced PPN populations, whereas other areas may be severely impacted by the increased spread of PPNs. As a result, PPN survival, reproduction, activity, and movement will be increasingly impacted by changes in:

##### 3.2.2.1 Temperature

As global temperatures rise, more favorable conditions will be created for nematode survival and reproduction. As noted by Evans and Perry (2009), PPNs exhibit varying optimal temperatures for activities such as feeding, hatching, reproduction, and survival (Evans and Perry, 2009). The rate of nematode development is temperature-dependent, with slower progression in cooler temperatures and faster growth in warmer soil temperatures (Tzortzakakis and Trudgill, 2005). This can lead to an increase in nematode populations and range expansion, as well as earlier emergence and longer activity periods. Elevated temperatures are also likely to exacerbate water stress symptoms in plants infected by nematodes, consequently impacting their nutritional health (Somasekhar and Prasad, 2011). On the positive side, higher temperatures tend to result in a higher proportion of males, which are generally less pathogenic or non-pathogenic to plants (Papadopoulou and Triantaphyllou, 1982). Elevated temperatures may also disrupt nematode survival mechanisms such as anhydrobiosis, overwintering, and egg diapause, which are vital against extreme environmental conditions (Evans and Perry, 2009). Some species may be inhibited by higher temperatures. For example, *Meloidogyne hapla* Chitwood, 1949 occurs only at higher elevations or latitudes related to temperature. Likewise, *Sphaeronema sasseri* Eisenback and Hartman, 1985 dies at room temperature but thrives at cooler temperatures which rarely exceed 15°C (Eisenback and Hartman, 1985).

##### 3.2.2.2 Precipitation

Changes in precipitation patterns can affect soil moisture levels, which can in turn impact nematode survival and activity. Increased precipitation can create more humid conditions that favor activity for certain PPNs (Decraemer and Hunt, 2006), reniform nematodes in particular (Dutta and Phani, 2023). These nematodes are pathogens of cotton, soybean, and most vegetables and tend to thrive in soils with a moderate amount of sand (Grabau, 2017). But increased humidity can also lead to increased fungal activity, known to kill nematodes acting as natural biocontrol agents. While drought conditions can reduce nematode survival by eliminating their plant hosts, at the same time, they can help in nematode reproduction given PPNs’ preference for sandy soil.

##### 3.2.2.3 Host plant distribution

Climate change can also impact the distribution and range of host plants, which can affect nematode populations. Changes in plant distribution can create new opportunities for nematode infestation, or reduce the availability of suitable hosts, which can lead to declines in nematode populations.

##### 3.2.2.4 Extreme weather events

Climate change is also expected to increase the frequency and intensity of extreme weather events, such as floods, droughts, and storms. These events can disrupt nematode populations and distribution by altering soil moisture, temperature, and other environmental factors and by increasing the probability of spreading PPNs via water, soil, and air.

##### 3.2.2.5 Changes in CO_2_ levels

Under conditions of increased CO_2_, it’s conceivable that nematodes might need to ingest more plant matter to sustain their population levels, owing to the diminished nitrogen content in plants. This could potentially lead to greater plant harm and decreased crop yields (Somasekhar and Prasad, 2011). Usually, however, since CO_2_ is a limiting factor in plant growth, increased levels of this gas may stimulate plant growth and make the plant more vigorous and able to sustain more nematodes without harm. Or, it may enhance the size of the root system which may enable nematode populations to increase beyond the capacity of the host.

Overall, the impacts of climate change on PPNs are complex and can vary depending on the nematode species, host plant, and environmental conditions. PPNs’ inability to disperse over long distances without a vector may help in forecasting their geographic distribution based on climate change models; however, with more research showing extensive areas of PPN infestations being attributed to environmental and human conditions increasing their migration to the food supply chain, maintaining plants in good health and early detection and mitigation are still considered to be the best approaches to addressing the variable and hard to predict distribution of PPNs triggered by climate change. Climate change is likely to have significant impacts on nematode survival, reproduction, distribution, and activity, with important implications for food security and safety. Significant supporting research remains to be completed.

## 4 Strategies for detecting, managing, and preventing the spread of nematodes in the food supply chain

According to the Department of Primary Industries and Regional Development’s Agriculture and Food, Western Australia, once nematodes are present, they are almost impossible to eliminate. As the saying goes, “Nematodes are forever”. Therefore, it’s imperative to focus on early detection and prevention of their spread. In many countries, the control of plant pests and diseases, including nematodes, is governed by *statutory regulations.* These regulations are enacted and enforced when the severity of the organisms or diseases justifies such actions (Southey, 1979). In the United States, the enforcement of these regulations falls under the purview of the APHIS, an agency dedicated to protecting the health and value of America’s agriculture and its natural resources.

The globalization of trade, both nationally and internationally, has led to a heightened introduction of invasive species, including nematodes, at an unprecedented rate. The International Plant Protection Convention (IPPC), recognized by the World Trade Organization’s Sanitary and Phytosanitary Agreement (WTO-SPS), is pivotal in developing international standards, guidelines, and recommendations for phytosanitary measures concerning food and agriculture-related trade (Haque and Khan, 2021). In adherence to the IPPC, member countries have established National Plant Protection Organizations (NPPOs), responsible for implementing Plant Quarantine (PQ) regulations. These regulations have public authority and aim to protect against the dissemination of invasive, injurious, and exotic pests and diseases (IPCC, 2021).

Today, the presence of PQ regulations is a global norm, with many countries combining these with Biosecurity protocols. Additionally, under the auspices of the WTO and governed by the Commission of Phytosanitary Measures (CPM), the International Standards for Phytosanitary Measures (ISPMs) are applied by WTO members under the WTO-SPS agreement to fulfill the requirements of the IPPC (Haque and Khan, 2021).

As outlined by Haque and Khan (2021), the ISPMs that apply to PPNs include specific quarantine treatments for imported commodities, prohibitions on the entry of hosts likely to carry particular nematodes, and guidelines on importing only plants with bare roots or those grown in approved media from nematode-free areas. Phytosanitary measures also encompass restrictions on the movement of soil and machinery from infested fields and encourage the immediate disposal of infected plant material and soil. If a contaminated material is detected at a point of entry, it is either recommended for deportation or destruction. Furthermore, upon the detection of a regulated or quarantine nematode, regulatory measures are taken to contain the spread.

Despite these stringent regulations, the movement of plants and produce continues to pose the risk of introducing nematodes into new environments. The increasing global movement of crops and food products facilitates the spread of nematodes, raising concerns about their introduction into non-native ecosystems (Tileubayeva et al., 2021). Even in developed economies, where strict regulations are in place for nurseries and greenhouses, these facilities remain risk factors for the spread of PPNs. The abundance of nematodes found in vegetable crops grown in greenhouses, coupled with their ease of spread through the movement of infested soil or plant material, presents a significant threat to the food supply chain (Tileubayeva et al., 2021). Important nursery nematodes include root-knot (*Meloidogyne* spp.), lesion (*Pratylenchus* spp.), foliar (*Aphelenchoides* spp.), and stunt (*Tylenchorynchus* spp.) nematodes. Burrowing (*Radopholus* spp.) and reniform (*Rotylenchulus* spp.) nematodes are also notable for injuring nursery crops and are subject to quarantine. The presence of the citrus nematode (*Tylenchulus semipenetrans*), for example, can disqualify a site for use as a citrus nursery (Crow and Dunn, 2005). Sanitation measures, awareness of visual symptoms, and, if necessary, the use of appropriate nematicides and containment measures can mitigate some risks. Typically, nursery and greenhouse management in developed economies necessitates specialized knowledge and adherence to stringent regulations. Conversely, in low- and middle-income countries, control measures are less rigorous, leading to situations where global trade facilitates nematode transmission. This problem has been further exacerbated by the increasing online availability and sale of live ornamental and horticultural plants in many countries (International Association for the Plant Protection Sciences (IAPPS), 2016).

Apart from global trade activities, environmental conditions and farming practices significantly influence the risk of PPN infestation in crops, posing a threat to both national and global food supplies. To counteract these threats, biosecurity measures play a critical role in preventing the introduction and spread of pests in crops and the wider food supply chain. These measures include quarantine, inspection, monitoring, and regulatory frameworks that limit the movement of crops and plant materials across borders.

Early, accurate and swift *detection and diagnosis* are paramount for effective nematode population management (Shao et al., 2023). Kantor et al. (2022) emphasize the necessity of ongoing sampling and scouting efforts to identify both regulated and emerging PPNs. The urgency for pest surveillance enables early detection and prevents the establishment and proliferation of new nematode threats, underscoring the need for specialized nematode diagnostic labs. While such facilities are available at many land-grant universities, a limited number provide nematode diagnostic services, potentially leading to a significant number of unreported cases. ISPMs offer guidance for pest surveillance through general surveillance, where information on specific pests is collected from various sources for NPPO use, or through specific surveys, where NPPOs collect information on pests of concern from particular sites over a defined period (Haque and Khan, 2021). ISPMs also provide protocols for the official diagnosis of regulated pests relevant to international trade.

The traditional morphology-based taxonomy of nematodes is complicated by intraspecific variation, but tools and techniques based on biochemical and molecular markers have been successful in diagnosing a wide array of nematode species (Carneiro et al., 2017). The transition from traditional morphological identification to advanced biochemical and molecular techniques, such as DNA barcoding and quantitative real-time PCR, has significantly enhanced the accuracy of PPN identification. Despite their effectiveness, these methods require specific equipment, and skilled personnel, and can be time-consuming and expensive for processing large sample volumes.

Isothermal amplification methods like LAMP (Loop-mediated Isothermal Amplification) and RPA (Recombinase Polymerase Amplification) are lauded for their simplicity and rapid application in the field, addressing some of these challenges. Furthermore, the integration of remote sensing, molecular techniques, and the advent of machine learning and artificial intelligence promise to further refine nematode detection processes, especially for handling large sample sizes efficiently (Akintayo et al., 2018; Shao et al., 2023). Recent advancements in deep learning have already shown promising results in the classification and detection of plant pests, achieving accuracy rates as high as 93% (Cheng et al., 2017; Xie et al., 2018; Kasinathan et al., 2021; Picek et al., 2022).

For instance, a novel approach by Agarwal et al. (2023) utilized a public dataset of annotated images featuring PPNs extracted from heterogeneous soil samples to develop automated identification methods via deep-learning object detection models. This technique, accommodating the variability found in real-world samples, including a mix of PPNs and non-plant parasitic species, marks a significant advancement from studies focused on isolated nematode genera in homogenous samples (Akintayo et al., 2018; Uhlemann et al., 2020; Qing et al., 2022; Shabrina et al., 2023). Although this method has its limitations, its potential for enhancing PPN identification in complex samples is substantial. The future direction involves integrating these innovative models with existing ones that target specific nematode genera, creating a more comprehensive and efficient diagnostic process (Agarwal et al., 2023).

No single solution is sufficient for PPN management. Multiple strategies are required, tailored to specific scales, geographies, and the systems and tools available. Effective management of nematode infestations necessitates a complex, multidirectional, and context-specific approach. It begins with foundational steps such as raising awareness and educating stakeholders about the significance of these pests. As nematodes can cause substantial yield losses across various crops, resulting in significant economic impacts, those involved in agriculture need to understand the threat posed by these organisms.

Despite significant discoveries in nematology since the 1950s and an early call (1994) for more research, education, and outreach in plant and soil nematology (Barker et al., 1994), the field has seen a narrowing of nematology experts due to limited job opportunities, even as the threat from PPNs has increased. In 1961, Thorne (1962) highlighted the necessity for trained nematologists, equating their importance to that of entomologists or plant pathologists in addressing world food problems. This statement remains relevant today, especially considering global climate change and the expanding threat posed by nematode pests. Correct identification of nematode species is essential for choosing appropriate control methods. Therefore, cultivating a future nematology workforce that can operate at the intersection of scientific innovation and agricultural extension work, involving *education, training, and outreach* to stakeholders, is of paramount importance. The field of nematology requires increased attention from federal and state authorities, as well as academic institutions, to develop career pathways that will nurture the next generation of nematologists (Kantor et al., 2022).

When the appropriate detection and diagnosis expertise and tools are in place, the next step involves understanding nematode *threshold numbers*. These thresholds represent the population density at which the cost of intervention balances out the economic damage caused by the pest (Ferris, 1978). Consequently, action below this threshold is not economically justified, as the expense of management would exceed the losses inflicted by the nematodes. Therefore, identifying the specific types of PPNs, their population densities, and evaluating their potential economic impact on crops is critical before formulating intervention plans (Hill, 1988).

Upon establishing the threshold, farmers and agricultural workers can consider a mix of traditional and new biosecurity measures.

### 4.1 Traditional biosecurity measures

Include using certified nematode-free planting material, practicing clean farming, rotating crops, managing soil moisture, controlling weeds, utilizing resistance, and applying soil fumigation and nematicides. Details on the critical practices for controlling PPNs are as follows:

#### 4.1.1 Use of certified nematode-free planting material

Start with planting materials that are certified to be free of PPNs. Utilizing nematode-resistant cultivars or plant varieties, which are less susceptible to nematode infestations, adds another layer of defense. This strategy can include planting resistant hybrids or grafting susceptible plants onto resistant rootstocks to reduce the risk of nematode introduction to fields.

#### 4.1.2 Good and clean farming practices

Regular cleaning of field/nursery or greenhouse equipment, including tillage equipment, planters, and harvesters, is crucial to prevent nematode spread. Washing with high-pressure water is usually the safest, cheapest, and best practice. Avoiding the transfer of soil from one place/field to another and ensuring cleanliness of boots, tools, and vehicles when moving between fields are important practices, as nematodes can survive for long periods in soil and easily spread, leading to potential soil contamination and increased crop damage risk. Farming operations should begin in an uncontaminated field and end in a field that has nematode populations.

#### 4.1.3 Crop rotation

This is one of the most effective strategies for managing PPN populations. Rotating different crops in the same field annually can prevent nematode population build-up. The method involves alternating crops susceptible to nematodes with non-host crops, preventing nematode populations from reaching harmful levels. Fallowing, which starves nematodes by removing their host crops, can also be effective, particularly when combined with conducive environmental conditions or anaerobic soil disinfestation techniques (Duncan, 1991). However, with sensitive crops or with persistent nematodes rotations of more than one year may be necessary to reduce the PPNs below damaging thresholds. The ancient Incas practiced a six-year rotation with the persistent potato cyst nematode because the population in only reduced by 10% each year.

#### 4.1.4 Soil moisture management

Since nematodes thrive in moist soil, controlling soil moisture can help reduce their populations. Likewise, some nematodes cannot tolerate flooded conditions, which can be used as a cultural control tactic (Bridge, 1996).

#### 4.1.5 Weed control

Weeds can act as hosts for nematodes, so controlling weed populations can decrease nematode populations and the risk of virus transmission because these weeds also serve as reservoirs of plant viruses (Dufus, 1971).

#### 4.1.6 Soil fumigation and nematicides

These are common techniques involving the application of chemicals to soil to kill nematodes (Giannakou and Panopoulou, 2019).

These traditional methods, while crucial, may not always be sufficient for early detection of PPN infestations, as symptoms may not appear until significant damage has occurred or a particular nematode species may not cause harm in its indigenous environment, but it may wreck havoc when it is introduce into a new habitat or plant host. Therefore, integrating these traditional approaches with newer technologies can enhance early detection capabilities and allow for more timely management decisions.

### 4.2 Emerging biosecurity measures

There is an increasing need to understand the geographic distribution and prevalence of certain PPNs, along with metadata containing PPN species and environmental vectors of transmission. This knowledge helps in advancing PPNs control strategies and preventing the spread of these pathogens. Climate change, with predictions of rising global temperatures and significant fluctuations in temperature and precipitation, is expected to influence the movement of PPNs northward or to higher altitudes, potentially intensifying their severity in regions where they are already present (Chakraborty et al., 2000).

Regular monitoring of fields can aid in the early detection of nematode infestations, allowing for timely intervention and improved decision-making. Unmanned Aircraft Systems (UAVs) are being employed as rapid and non-destructive methods to capture multispectral images of plants to uncover levels of stress and, more challengingly, the potential causes and spread of disease (Mogili and Deepak, 2018; Barbedo, 2019; Raparelli and Bajocco, 2019; Tsoulias et al., 2023). Spectral imaging, a technique used to capture and analyze the spectrum of light reflected, transmitted, or emitted by an object, combines imaging and spectroscopy to obtain both spatial and spectral information. This technology, initially developed for remote sensing applications and earth observations via satellites (Goetz, 1995), has evolved significantly over the past few decades. The development of hyper- and multispectral instruments mounted on aerial vehicles (e.g., drones or helicopters) and ground-based systems (e.g., tractors and agricultural implements) has progressed alongside rapid advances in electronics, hardware, software, and computing power. These systems are now widely used in various areas of farming to obtain higher temporal and spatial resolution of sensing images, enabling precision agriculture (Tsoulias et al., 2023).

The methodologies for image collection are well-established, involving flying over fields and capturing images multiple times during the growing season. UAVs are now affordable, low-cost, and relatively easy to maneuver, allowing farmers, including small-scale ones, to obtain crop information quickly and with acceptable accuracy (Cavalcanti et al., 2023). However, it is advisable to consult with experts in remote sensing and unmanned aircraft systems, such as extension agents or certified professionals, to ensure appropriate setup, as factors like flight height, camera resolution, and calibration, as well as weather conditions, can impact image quality (Cavalcanti et al., 2023).

The post-collection image analysis is more labor-intensive and, especially for larger areas, requires computer programming skills, the appropriate selection of indices (e.g., NDVI, NDRE, GNDVI, EVI, SIPI) that best relate to the targeted nematode infection type (Brown, 2023; Kalinzi, 2023), data wrangling, and change-detection analysis. Despite significant advances in these technologies, achieving broadband coverage can be challenging in rural areas where farming predominantly occurs. When establishing thresholds, farmers must consider broadband coverage and associated costs. For example, the Pennsylvania Broadband Map, which represents FCC data in terms of broadband service, assists in estimating project costs for providing service to locations not having broadband services as defined by the FCC (PennState Extension Pennsylvania Broadband map). The map includes reserve prices, eligible sites, existing structures, transmission lines, substations, towers, and legislator information, as well as measuring tools for assistance (PennState Extension).

Research showcasing the applicability and value of using remote sensing coupled with machine learning to monitor nematode-caused diseases in crops has been increasing in recent years. Kalinzi (2023) demonstrated that multispectral drone remote sensing is a robust and promising approach for identifying levels of soybean cyst nematode infestation at the plot level. In another study, Cavalcanti et al. (2023) used aerial images collected by a low-cost unmanned aerial vehicle, coupled with ground-truthing (field measurements and nematode egg extractions from infected plants’ roots), to gather information on lettuce crops to estimate root-knot nematode incidence. The authors calculated lettuce vegetation cover by focusing on changes in lettuce growth in the field through RGB images, yielding consistent results with field measurements. While successful in estimating changes in plant growth, the color indices from RGB images were mainly used to segment plants from the overall image without comparing field RGB images due to high field luminosity limitations and the impossibility of calibrating it in low-cost RGB cameras. Other research using RGB imaging in greenhouse settings was more successful in calculating vegetation indexes and estimating nitrogen and chlorophyll content in greenhouse tomatoes (Mercado-Luna et al., 2010), rice leaves (Saberioon et al., 2014), and lettuce (*Lactuca sativa* L.) crops (Odabas et al., 2017), due to better luminosity calibration compared to field studies.

When PPN management methods have failed or are not feasible, the goal of *control* is to reduce the population density and abundance below the economic threshold level. Chemicals and biological controls are the most effective and rapid means of controlling pests. While chemicals are toxic and hazardous and less cost-effective but have a quicker reaction, biological controls combined with integrated pest management (natural control, employing principles of control in regulation, prevention, avoidance, eradication, protection, therapy, and resistance, selection of control tactics on various parameters, and application of all these principles in all decision-making processes) should be considered when cost, sustainability, and ecological factors are of primary importance (Haque and Khan, 2021).

These mixed practices, grounded in a thorough knowledge of nematode behavior and crop susceptibility, as well as technological advances, are pivotal in sustainable nematode management and crop protection strategies.

## 5 A framework to synthesize the biosecurity risks of PPN on human food supply

See **Figure 2**.

**Figure 2:**
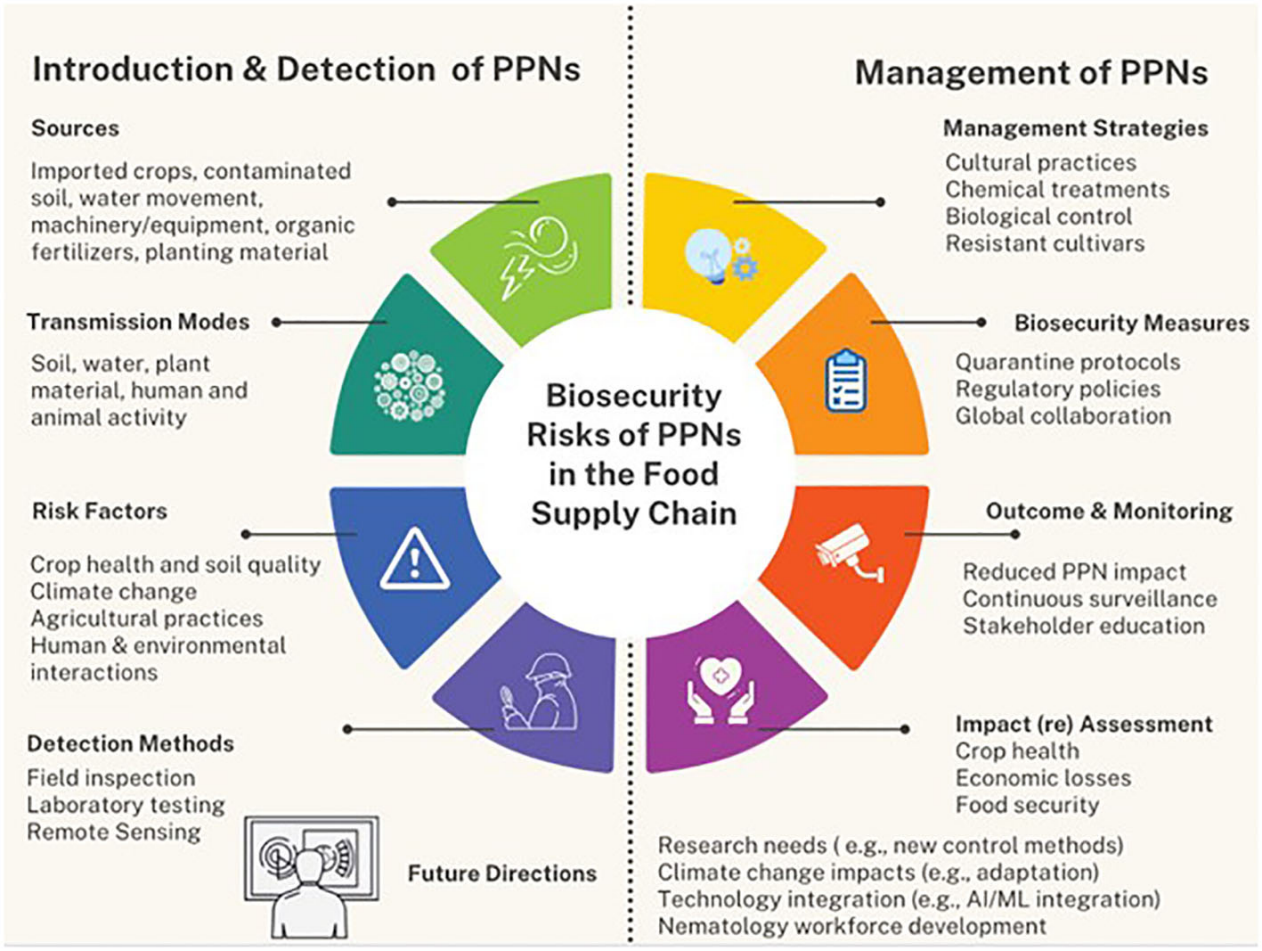
Flowchart illustrating the framework for synthesizing the biosecurity risks of PPNs on the human food supply. This figure encompasses the introduction of PPNs, biosecurity risks, impact on food safety and security, detection and management strategies, recommendations for future research, and the desired outcomes.

## 6 Conclusion

This review highlights the critical importance of biosecurity to agriculture, focusing on the significant threat posed by PPNs to crop health and, consequently, to food security. PPNs are not just agricultural pests; their impact extends to the entire food supply chain, underlining their role as a central concern in global food safety and security. Impacted by climate change and a more complex and expanding national and global trade, the risk of PPNs being introduced to new environments increases as well, thereby exacerbating their impact. The introduction of just one roque species has the potential to wipe out an entire species and cause incalculable environmental and economic harm to an entire continent. This article has outlined the various aspects of PPNs, including their transmission modes, factors increasing the risk of infestation, and their impact on crops and food safety. It emphasized traditional and emerging strategies for detecting, managing, and preventing the spread of nematodes, highlighting the necessity of early detection and prevention due to the near impossibility of eliminating PPNs once they have become established. The role of statutory regulations and international standards in managing these biosecurity risks was also discussed.

PPNs pose a direct threat to crop yields and quality, which can lead to substantial economic losses and jeopardize food security. Infestations can also exacerbate the risk of microbial contamination, compromising food safety. Therefore, effective management and control of PPNs are essential to ensure the integrity of the food supply chain and to protect global food security. Future research should focus on several key areas: The development of more advanced and accessible diagnostic tools for early detection of PPNs, integrating technologies such as artificial intelligence and remote sensing; Enhanced understanding of the impact of climate change on PPN distribution and behavior, which is crucial for predicting future risks and developing appropriate strategies; Exploration of sustainable and eco-friendly pest management practices, such as integrated pest management, to reduce reliance on chemical controls and; Expansion of global nematology expertise, emphasizing education and training to build a robust network of professionals capable of addressing PPN challenges. Further research into the socioeconomic aspects of PPN management, particularly in developing countries, to ensure that strategies are not only effective but also equitable and sustainable should also be considered.

In conclusion, tackling the challenge posed by PPNs requires a multifaceted approach, blending traditional methods with cutting-edge technologies, supported by a strong foundation of research, education, and international collaboration. This approach will not only safeguard crop health but also ensure the stability of our global food supply within changing climates.

## Author contributions

CK: Writing – original draft, Writing – review & editing, Conceptualization, Formal analysis, Investigation, Methodology, Project administration, Software, Visualization. JE: Writing – review & editing, Data curation, Validation. MK: Writing – original draft, Writing – review & editing, Conceptualization, Data curation, Investigation, Methodology, Project administration, Visualization, Resources.
